# FGFR- gene family alterations in low-grade neuroepithelial tumors

**DOI:** 10.1186/s40478-020-00898-6

**Published:** 2020-02-21

**Authors:** Tejus A. Bale

**Affiliations:** Department of Pathology, Memorial Sloan Kettering Cancer Center, 1275 York Street, New York, NY 10065 USA

**Keywords:** Low grade glioma, Low grade glioneuronal tumor, FGFR-fusion, FGFR-mutant

## Abstract

The discovery of fibroblast growth factor receptor (FGFR) gene family alterations as drivers of primary brain tumors has generated significant excitement, both as potential therapeutic targets as well as defining hallmarks of histologic entities. However, FGFR alterations among neuroepithelial lesions are not restricted to high or low grade, nor to adult vs. pediatric-type tumors. While it may be tempting to consider FGFR-altered tumors as a unified group, this underlying heterogeneity poses diagnostic and interpretive challenges. Therefore, understanding the underlying biology of tumors harboring specific FGFR alterations is critical. In this review, recent evidence for recurrent FGFR alterations in histologically and biologically low-grade neuroepithelial tumors (LGNTs) is examined (namely *FGFR1* tyrosine kinase domain duplication in low grade glioma, FGFR1-TACC1 fusions in extraventricular neurocytoma [EVN], and FGFR2-CTNNA3 fusions in polymorphous low-grade neuroepithelial tumor of the young [PLNTY]). Additionally, FGFR alterations with less well-defined prognostic implications are considered (*FGFR3-TACC3* fusions, *FGFR1* hotspot mutations). Finally, a framework for practical interpretation of FGFR alterations in low grade glial/glioneuronal tumors is proposed.

## Introduction

The search for disease-defining genetic alterations in brain tumors has characterized the last several decades in neuropathology: one particularly exciting arena has been the discovery of a host of fibroblast growth factor receptor (FGFR) gene family alterations as apparent drivers of primary brain tumors. However, this particular group of lesions has proven especially challenging as they are not confined to either high or low grade, nor to adult vs. pediatric lesions. In fact, FGFR alterations are implicated across a host of human cancers, promoting oncogenesis as a result of overexpression, amplification, mutations, and structural variations [28, 35, 51, 73].

The FGFR family consists of four highly conserved transmembrane tyrosine kinase receptors (*FGFR1–4*) and represents a fundamental receptor tyrosine kinase (RTK) signaling pathway. FGFRs dimerize in the presence of any of 22 known ligands, triggering downstream signaling pathways well-implicated in tumorigenesis; these include the mitogen activated protein kinase (MAPK) and phosphoinositide-3-kinase (PI3K)/Akt pathways among others [14, 20, 34, 45]. Beyond playing an important role in CNS embryonal development, FGFR signaling influences angiogenesis and tumor cell migration, differentiation, proliferation, and survival. Not surprisingly, FGFRs have emerged as a major target for cancer therapeutics across tumor types and multiple targeting strategies are under investigation [5, 13, 16, 19, 24, 30, 47, 48].

The optimal use of targeted therapy in brain tumors remains under investigation, and its efficacy in low-grade tumors, which would conceivably be slow growing, has been difficult to assess [72]. Although detection of these possible therapeutic targets is of great clinical interest, high quality clinical data remains limited. Ahead of this, understanding the biological implications of specific FGFR alterations, and how this relates to tumor subclassification, is paramount; this is particularly true among histologically low-grade tumors.

Recently the Consortium to Inform Molecular and Practical Approaches to CNS tumor Taxonomy-Not Official WHO (cIMPACT-NOW) released update 4, which specifically addressed so-called “pediatric-type diffuse gliomas” [22]. In contrast to the IDH*-* wild type, diffuse gliomas encountered in adults, diffuse gliomas in children and adolescents most commonly harbor a different constellation of mutations and fusions including alterations in *FGFR1* [56, 77]. The guidelines recommend distinguishing these from adult-type tumors to provide more accurate prognostication, and in some instances guide therapy; delineating relevant diffuse gliomas as harboring either tyrosine kinase domain duplication (TKDD) or single nucleotide variants in *FGFR1*. This is an important step in brain tumor classification and more accurately reflects the relatively prolonged disease course and better overall survival of these pediatric lesions, certainly when compared to IDH-wild type, “adult” tumors. However, while it may be tempting to further consider FGFR-altered tumors as a unified group, there remains significant heterogeneity among them.

**Fig. 1:**
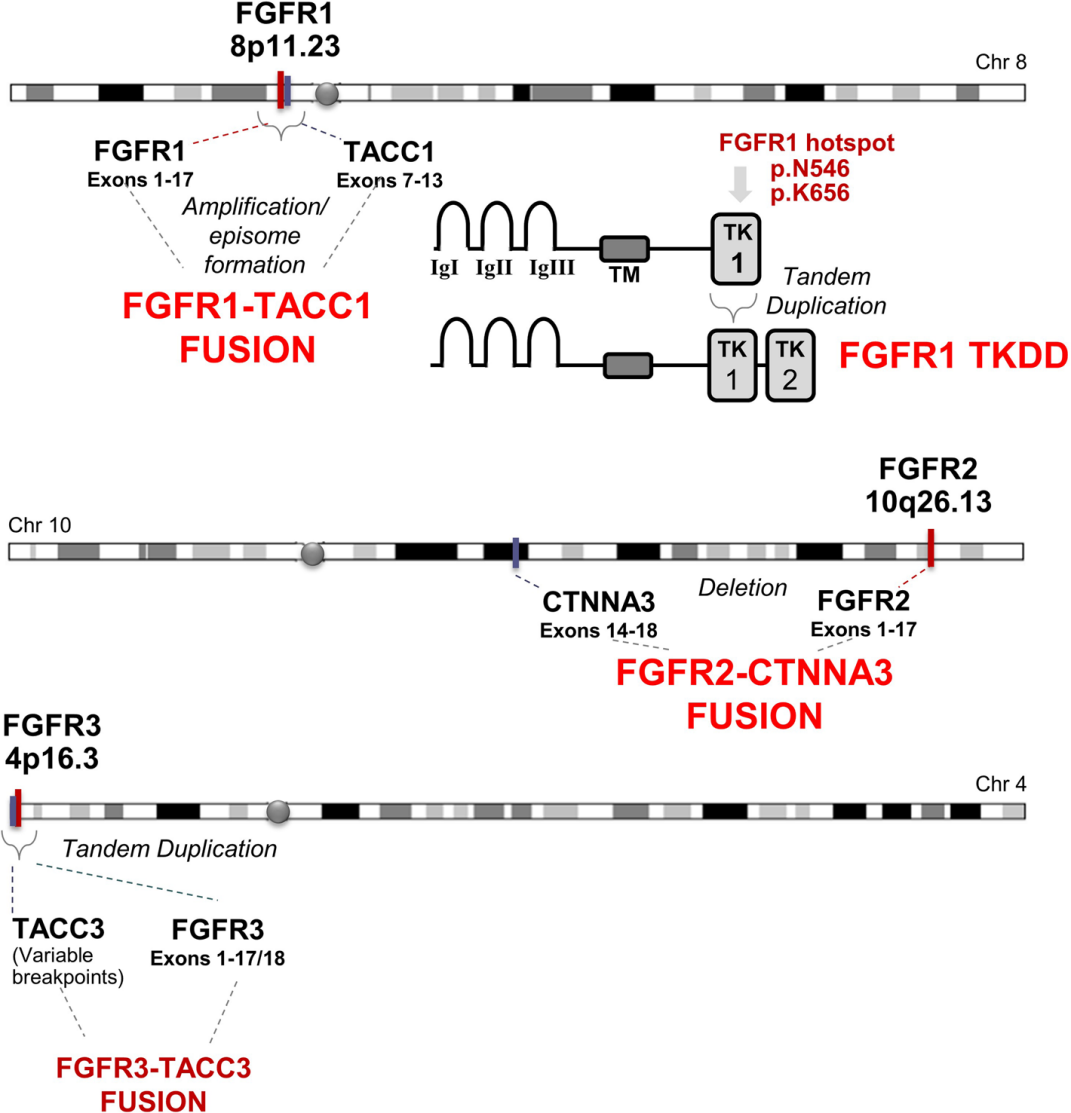
Summary of common FGFR alterations in brain tumors. Some alterations are strongly associated with low grade neuroepithelial lesions: *FGFR1*-TKD, *FGFR1-TACC1* fusion, *FGFR2-CTNNA3* fusion. Others (including *FGFR1* hotspot mutations and *FGFR3-TACC3* fusions) are described in low-grade as well as high-grade tumors, requiring cautious interpretation when encountered in histologic LGNTs

In this review, recent evidence for recurrent FGFR alterations in histologically and biologically low grade neuroepithelial tumors (LGNTs) is examined. These include *FGFR1* tyrosine kinase domain duplication in low grade glioma, *FGFR1-TACC1* fusions in extraventricular neurocytoma (EVN), and *FGFR2-CTNNA3* fusions in polymorphous low grade neuroepithelial tumor of the young (PLNTY). Additionally, FGFR alterations with less well-defined prognostic implications are considered (*FGR3-TACC3* fusions, *FGFR1* hotspot mutations). The structure of these alterations is summarized in Fig. 1. Finally, a proposed framework for interpreting the implications of specific FGFR alterations regarding tumor subclassification and prognostication is presented.

### Genotypic-phenotypic correlations in low grade lesions with FGFR alterations

Emerging evidence has demonstrated that certain low-grade histologic entities appear to be dominated by specific FGFR alterations. While these mutations have not yet been raised to the level of definitional characteristics by the WHO (and are therefore not required for rendering a diagnosis), there remains (with rare exceptions), a virtual absence in the reported literature of associated high-grade histology and/or aggressive clinical behavior in association with select FGFR alterations. As such, by and large, these alterations may be reasonably regarded as hallmarks of the following low grade neuroepithelial tumors.

#### FGFR1- tyrosine kinase domain duplication (FGFR1-TKDD) in low grade glioma (LGG)

**Fig. 2:**
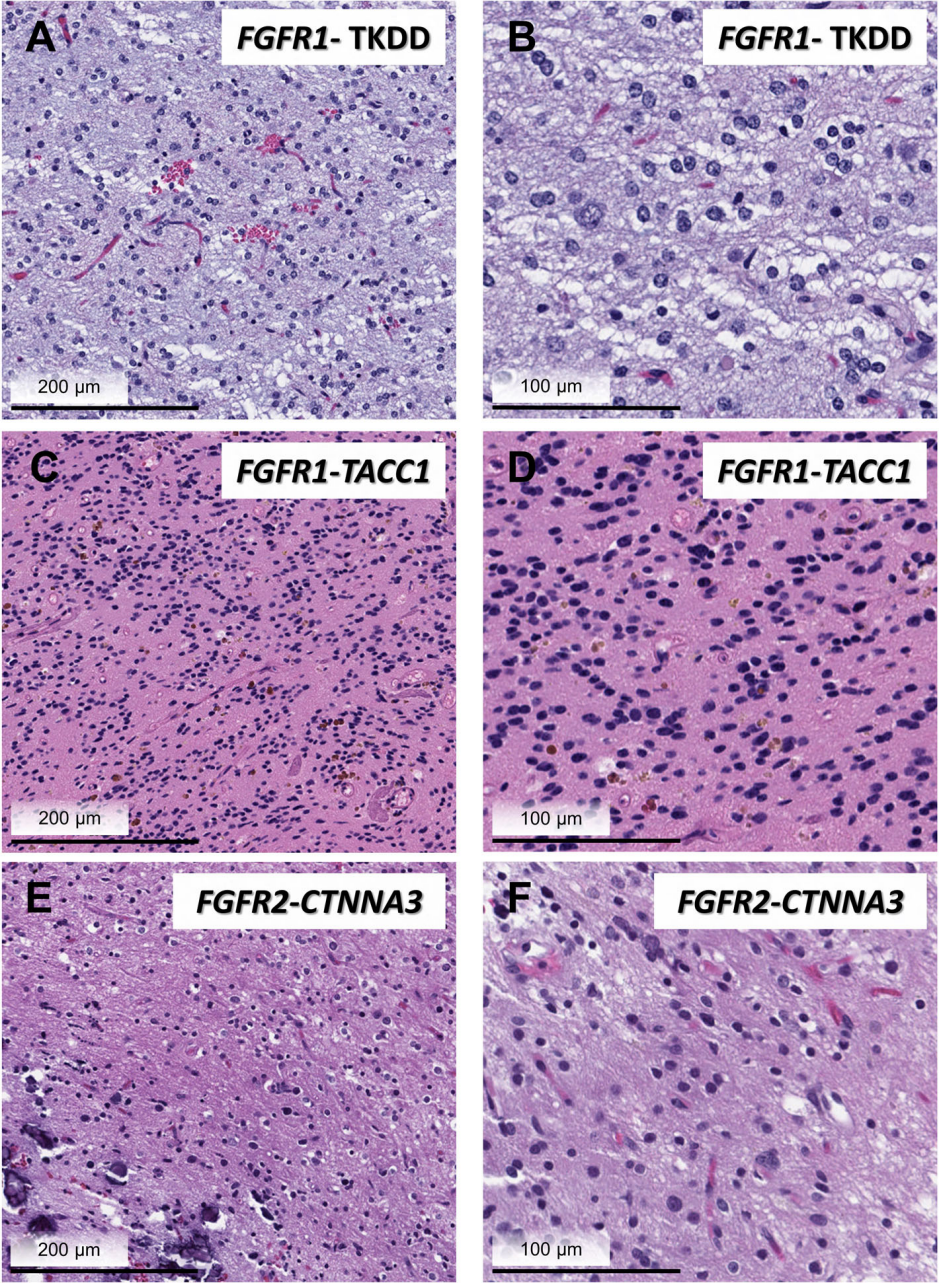
Histologic features of FGFR-altered LGNTs. Three examples of LGNTs bearing characteristic FGFR-alteration are shown: DNET with *FGFR1*- TKD (**a**, **b**), EVN with *FGFR1-TACC1* fusion (**c**, **d**), and PLNTY with *FGFR2-CTNNA3* fusion (**e**, **f**). Note that while histologic features of each lesion met diagnostic criteria in keeping with a specific entity, LGNTs share many overlapping histologic features including bland neurocytic/ oligodedroglioma-like nuclear features and of lack of significant proliferative or mitotic activity

Among the most important insights gained from landmark sequencing studies examining the molecular landscape of pediatric low grade glial and glioneuronal tumors was the identification of an intragenic duplication of the entire *FGFR1* region encoding the tyrosine kinase domain (TKD). This duplication includes exons 10–18 and produces an in-frame fusion separated by a linker element of variable length [56, 77]. Histologically, lesions harboring FGFR1-TKDD appear to be predominately diffuse gliomas located in the cerebral cortex. Duplication of the FGFR1 TKD has also been reported in low-grade astrocytomas more suggestive of other specific histologic entities including pilocytic astrocytoma (typically extracerebellar) and dysembryoplastic neuroepithelial tumor (DNET, Fig. 2a, b) [23, 37, 40, 60, 77].

While encompassing a significant subset of LGNT (7.4–24%), this alteration appears. to be virtually absent in high-grade gliomas (HGG) [38, 77]. In the original report, a cohort of 33 HGG were screened for duplication of the *FGFR1* region encoding the TKD, revealing only one tumor (diagnosed as anaplastic oligoastrocytoma, WHO grade III) that had progressed from a grade II tumor. No FGFR1-TKDD positive cases were detected in adult-type oligodendrogliomas, IDH-mutant and 1p/19q co-deleted [77]. Since then, the association of FGFR1-TKDD with anaplastic histologic features has proven to be an exceedingly rare phenomenon. One reported case of a rosette forming glioneuronal tumor (RGNT) having focal DNET-like features exhibited multiple local recurrences over a ten-year period, ultimately demonstrated elevated mitoses and high-grade histology, and was shown to harbor FGFR1-TKDD in addition to a frameshift mutation in *ATRX* [33]. Additionally, a glioneuronal tumor with features of pilocytic astrocytoma and pleomorphic xanthoastrocytoma also harboring FGFR1-TKDD was reported to demonstrate focally elevated mitotic activity; molecular characterization revealed multiple additional variants of unknown significance [3]. It is noteworthy in this instance that, while histologic criteria for anaplasia were met, without long term follow-up data, the biologic and prognostic significance of these findings are unclear. Excepting these rare instances, FGFR1-TKDD has been associated with tumors manifesting bland histology and benign clinical behavior.

#### FGFR1-TACC1 fusion in extra ventricular neurocytoma

Among the most highly recurrent chromosomal translocations across human cancers are those involving fusions of FGFR genes with members of the purportedly oncogenic TACC protein family (*TACC1*, *TACC2*, and *TACC3* [21, 52, 75]). TACC proteins contain a coiled-coil domain at the C-terminus (TACC domain), which facilitates localization of the fusion protein to the centrosome and mitotic spindle [36, 53] in tum promoting aneuploidy and tumorigenesis [49, 69]. Constitutive FGFR activity and downstream MAPK/PI3K/mTOR pathway activation also results from the fusion [32, 43].

It is important to note that the highest frequency of FGFR-TACC chromosomal translocations is in HGG, namely IDH-wild type GBM where the fusion is between *FGFR3* and *TACC3*, located 48 kb apart on chromosome 4p16 [18, 50, 69] see FGFR3 fusions). Among FGFR-fusion positive glioblastomas, much less frequently encountered are FGFR fusions other than *FGFR3-TACC3*, including *FGFR1-TACC1* [18, 69, 70]. Homologous with regards to respective chromosomal locations, *FGFR1* and *TACC1* are located on chromosome 8p11; the molecular mechanisms with regards to downstream MAPK pathway activation as a result of *FGFR1-TACC1* fusion are also thought to be similar to those of *FGFR3-TACC3*, though less extensively studied and modeled [44].

In sharp contrast to *FGFR3-TACC3*, *FGFR1-TACC1* appears to more commonly associated with low-grade histology and biology, being especially prevalent in the context of extra ventricular neurocytoma (EVN). EVN is a rare primary brain tumor occurring within the parenchyma, outside the ventricular system. While a range of histopathological features may be encountered in EVN, these tumors generally resemble central neurocytoma (Fig. 2c, d). Not surprisingly, accurate diagnosis is confounded by overlapping morphological features with other LGNT entities. DNA methylation-based analysis of a cohort of EVN found that while a subset of histologically diagnosed EVN could be regrouped with other defined, established entities, a large fraction formed a clearly distinct, separate epigenetic group. Importantly, copy number analysis and RNA sequencing demonstrated *FGFR1-TACC1* fusion as a recurrent feature within the EVN methylation group (60%), in addition to a small number of other FGFR rearrangements (*FGFR3-TACC3, FGFR1-EVI5*) [67].

Indeed, many of the earlier descriptions of EVN predate newer molecular classification of brain tumors and may have been confounded by histologic overlap with other entities. The relationship between rare cases described as *FGFR1-TACC*1 fusion-positive HGG/GBM and cases of so-called “atypical EVN” with necrosis, vascular proliferation, and/or elevated mitotic activity, is unclear [25, 29, 41, 44, 69]. The majority of EVNs are well-differentiated and generally benign [11]. In the absence of elevated proliferative rate/mitotic activity, and particularly after complete resection, the rate of recurrence is low [25, 41]. While definitive grading criteria have yet to be established and survival data studied in additional independent cohorts, EVN corresponds histologically to WHO grade II, which is in keeping with reported survival data in molecularly-defined EVN, including those bearing *FGFR1-TACC1* fusions [67].

#### FGFR2- fusion (FGFR2-CTNNA3) in PLNTY

A recently described entity, “polymorphous low-grade neuroepithelial tumor of the young” or “PLNTY”, has been shown to harbor molecular abnormalities involving the MAPK pathway, including FGFR genes, and a unique fusion involving *FGFR2* [39]. These tumors, while morphologically somewhat variable, are characterized by infiltrative growth, oligodendroglioma-like cytologic features, and frequent calcification (Fig. 2e, f). Strong cluster of differentiation 34 (CD34) immunohistochemical expression has also been described. Belonging to a group of epilepsy associated low-grade neuroepithelial tumors in children and young adults, PLNTYs appear to have a predilection for the superficial cerebral hemispheres (particularly the temporal lobes), in keeping with prior reports of “long-term epilepsy associated tumors (LEATs)” [10, 39]. Most importantly, all indications point to the indolent behavior of PLNTY [9, 31, 37, 71].

In the original description by Huse et al. (2017) a novel fusion transcript was identified among the series of PLNTY, wherein *FGFR2* (including the kinase domain) joined with exons 14–18 of *CTNNA3* (to include the entirety of its C-terminal dimerization domain) [37, 58]. The oncogenic fusion is thought to result in homodimerization and autophosphorylation of FGFR2 and downstream MAPK/PI3K/mTOR pathway activation, similar to other FGFR fusions as previously discussed [15, 69, 71]. Molecular profiling of PLNTYs has demonstrated that they carry a distinct DNA methylation signature, suggesting that they are in fact a distinct biologic entity among at least a subset of LGNTs, including previously described “pediatric oligodendrogliomas” [56, 77]. No reports of *FGFR2-CTNNA3* fusion in association with a high-grade or aggressive tumor have been made to date. However, it is important to note that while *FGFR2-CTNNA3* appears to be relatively specific signature of PLNTY, the molecular landscape of PLNTY includes genetic abnormalities involving either *BRAF* or even *FGFR3*. These other alterations are not unique to PLNTY, and inasmuch as they are frequently also encountered in higher grade entities, should not be regarded as diagnostic of this entity or as predictive of a benign clinical course.

### Other FGFR alterations: unclear implications in LGNT

Several other alterations in FGFR genes have been reported in association with LGNTs, but their distribution is not limited to tumors with low grade histology or benign behavior. Therefore, the implications of these alterations in isolation are less clear. Cautious interpretation is advised, particularly in settings where infiltrating or undersampled tumor is a possibility.

#### FGFR3 fusions

The reality is that the implications of *FGFR3* fusion are clear: as previously stated, *FGFR3* fusions, most commonly *FGFR3-TACC3*, are by and large a feature of IDH-wild type glioblastoma, WHO grade IV [18]. Although FGFR-fusion positive GBM constitutes a small subset of GBM as a whole (~ 3%), the sheer preponderance of GBM relative to other types of glioma renders this the most common scenario in which *FGFR3* fusions will be encountered in most neuropathology practice settings [7, 18, 69].

Difficulty arises when this genetic feature of GBM is encountered in lower grade histologic entities. Detection of *FGFR3* fusions in histologically low-grade tumors is well-documented [18, 27, 37, 38, 77]. However, many of these cases were not been reported with sufficient long-term follow up to determine their clinical biology. This is not to say that *FGFR3* fusions cannot be associated with benign histologic entities; the sole *FGFR3-TACC3* fusion positive case in the original series of PLNTY for example was devoid of any high-grade features suggestive of GBM and demonstrated no evidence of disease or seizures after an extensive interval (89 months) [37]. Of note, *FGFR3-TACC3* fusions in GBM characteristically arise in order individuals, with frequent co-mutation of *TERT* promoter and loss of *CDKN2A/2B*, features that should help distinguish these cases from true LGNT, including PLNTY.

*FGFR3-TACC3* fusion gliomas, both low and high grade, exhibit characteristic histologic features, including monomorphous oligodendroglioma-like nuclei, “chicken-wire” capillary networks, and frequent microcalcifications [7]. While this may be reflective of the common end-result of FGFR fusions in all tumors (namely enhanced downstream signaling through MAP kinase pathway effectors), the histologic similarities suggest the possibility of *FGFR3*-fusion positive GBM arising from lower-grade precursor lesions. To date, however, there has been insufficient evidence to support this, and the relationship between high and low grade *FGFR-* fusion positive tumors, if any, remains unclear. Rather, *FGFR3* fusions should prompt a careful evaluation of clinical and neuroradiologic features and call for close surveillance following surgery, when encountered in an ostensibly LGNT.

#### FGFR1 hotspot (N546 & K656) mutations

Another frequently reported FGFR alteration among LGNTs is mutation of two hotspot residues (N546 & K656) in the tyrosine kinase domain of *FGFR1*, well-known to be activating and oncogenic [6, 46, 57, 76]. These two residues are the most commonly mutated residues in *FGFR1* in human cancers and interestingly are described predominately in CNS tumors, mostly histologic pilocytic astrocytomas [40, 78]. Somatic hotspot and germline mutations in *FGFR1* have also been implicated in the pathogenesis of DNET [60]. Of note, encephalocraniocutaneous lipomatosis (ECCL) a sporadic neurocutaneous syndrome with features of disordered RAS-MAPK signaling, appears to be mediated in at least a subset of cases by these very *FGFR1* mutations (in mosaic, somatic distribution) and also carries an increased risk of low-grade gliomas, again predominately of pilocytic astrocytoma histology [6, 8, 42, 54, 64]. It is emerging however, that while these *FGFR1-*mutant tumors certainly can be described histologically and biologically as low grade, they are distinct from typical pilocytic astrocytoma (WHO grade I), which are predominately driven by *BRAF* fusions. In fact, in some early studies, *FGFR1* mutation in pilocytic astrocytoma was associated with a significantly poorer prognosis, although sample size was small [4]. While no specific differentiating histologic criteria have been reported, it has emerged that there are distinguishing clinicopathologic features of these tumors; subsequent larger studies have revealed that pilocytic astrocytoma with *FGFR1* mutation are predominately extracerebellar and frequently midline in location, (in contrast to *BRAF*-fusion positive pilocytic astrocytomas, which predominate in the cerebellum) [40]. At the same time, hotspot *FGFR1* mutations have also been observed in adult and pediatric HGG, at the level of GBM (WHO grade IV) [12, 40, 57]. Notably, *FGFR1* hotspot mutations have been detected in up to 18% of adult midline glioma with high grade histology [55]. These *FGFR1*-mutant HGG frequently demonstrated a recurrent mutational profile in which H3 alterations (H3F3A K27M) and somatic mutations in *NF1* [40] were detected. Although this profile can be seen in tumors histologically equivalent to pilocytic astrocytoma, the underlying molecular features are strongly suggestive of biologic overlap with diffuse midline glioma, H3 K27M-mutant (WHO grade IV) [40, 65].

*FGFR1* hotspot mutations have also emerged as a molecular hallmark of rosette-forming glioneuronal tumor (RGNT) [26, 66]. RGNTs predominately affect young adults and are relatively rare neuroepithelial tumors with distinctive histologic features namely, the presence of neurocytes in rosettes or perivascular pseudo-rosettes in addition to an astrocytic component resembling pilocytic astrocytoma. It is on the basis of histology that the diagnosis is rendered. While in recent studies *FGFR1* hotspot mutations were invariably detected among RGNTs [66], their presence is not currently required for the diagnosis, (and as previously discussed, is certainly not unique to RGNT). Moreover, while RGNT corresponds histologically to WHO grade I and is generally considered benign, dissemination and progression have been reported in rare instances [1, 2, 62, 68, 74]. Of note, frequent co-mutation with *PIK3CA* as well as *NF1* have been reported in RGNT [66]. Mutation of PI3K pathway genes has been associated with aggressive clinical behavior in LGNTs, although further study is needed to determine their prognostic value in RGNT [26, 61]. On the whole, while there is clearly a role for *FGFR1* hotspot mutations in the pathogenesis of LGNT, their specificity for low grade histology and clinical behavior is highly dependent on histologic features and broader molecular context.

##### Practical approaches to FGFR alterations in LGNT

Based on available evidence, it appears that some FGFR alterations are more tightly correlated with specific histologic entities among LGNTs, while others may be encountered among variable tumor types, spanning histologic grades and clinical behavior. This poses significant challenges for molecular pathologists, neuropathologists and clinicians: how to determine which amongst these lesions are truly low grade, versus those with increased biologic potential. A practical approach to consider when encountering and “triaging” FGFR alterations in LGNT should involve determining 1) the presence of any atypical features and 2) the presence of additional molecular alterations. Atypical features worth noting in LGNT include both histologic and clinical features. For example, elevated mitotic activity, proliferation indices, and other indicators of high-grade histology should always be noted, even if only focally present in tumors bearing the FGFR alterations described herein. While definitive grading criteria await establishment, in general, bona fide LGNTs are not expected to display significant mitoses, necrosis, or vascular proliferation; proliferative indices would not be expected to exceed 1–2%. Similarly, a multidisciplinary clinical view should be given due consideration in these instances; atypical neuroimaging, and unusual clinical setting (i.e. PLNTY in an older individual [9, 59]) could potentially serve as important indicators of the true nature of the lesion.

By and large, FGFR alterations in LGNTs appear to be a reassuring finding, particularly when they are present in an otherwise genomically quiet background. Most LGNTs appear to be driven by a single molecular pathway, and typically by a single driver genetic alteration [56, 77]. This can be a challenge to definitively determine when taking a minimalist molecular diagnostic approach. Although next-generation sequencing may not be possible to perform in every case, determining the absence of additional alterations (loss of *CDKN2A/2B*, *TERT* promoter mutation, H3- mutation etc.) may be critical to determining the nature of the FGFR-alteration bearing tumor and broader genomic testing should be strongly considered [22].

## Conclusion

While for the purpose of this review, the role of FGFR alterations has been described in relation to specific histologic entities, the reality is that there is significant overlap of histologic features amongst LGNTs (Fig. 2). Although there is utility to the genotypic-phenotypic association between FGFR-alteration and tumor type, it may be more accurate to consider FGFR-altered neuroepithelial lesions as spanning a histologic spectrum. That this group also includes higher grade tumors implies that the spectrum is a biological one as well. Furthermore, it is important to bear in mind that FGFR- altered tumors are an important subset of a larger group of glial/glioneuronal tumors that are primarily driven by altered MAPK signaling [17, 37, 52, 71].

As previously noted, oncogenic FGFR signaling appears to play a role in a variety of cancer types, including extraneural tumors; FGFR pathway inhibition as a therapeutic strategy remains an area of active investigation. As clinical trials of FGFR inhibitors in brain tumors are ongoing or only recently completed (NCT01975701, NCT028224133, NCT02052778, NCT01948297), we have yet to fully explore the efficacy of this therapeutic approach. Recently, for example, a study found that FGFR inhibitors (AZ4547, dovatinib, PD173074, ponatinib) were more effective in reducing the growth of pediatric diffuse midline glioma, H3 K27M-mutant (diffuse intrinsic pontine glioma, DIPG) cells in vitro compared to Temozolomide [63]. However, much about the role of FGFR inhibitors in treatment of brain tumors, LGNTs in particular, remains to be understood. Optimal design of clinical trials and interpretation of data will be directly dependent on accurate classification of tumors bearing these FGFR alterations.

The complexity of FGFR signaling means that more research will also be necessary to better understand how FGFRs contribute to cancer biology beyond tumor initiation. The role of FGFRs in disease progression as well as associated mechanisms of treatment resistance remain largely unknown (but are certainly relevant issues in the treatment of low grade tumors). With advancing knowledge, we will continue to more accurately identify and stratify LGNTs based on their underlying molecular features, increasingly guiding therapeutic decisions now and in the imminent future.

## Data Availability

Data sharing is not applicable to this article as no datasets were generated or analysed during the current study.
